# A comparison of stage-specific all-cause mortality between testicular sex cord stromal tumors and germ cell tumors: results from the National Cancer Database

**DOI:** 10.1186/s12894-020-00609-2

**Published:** 2020-04-17

**Authors:** Kyle B. Zuniga, Samuel L. Washington, Sima P. Porten, Maxwell V. Meng

**Affiliations:** 1 Department of Urology, University of California, San Francisco, 550 16th Street, 6th Floor, San Francisco, CA 94158 USA; 2 Osher Center for Integrative Medicine, University of California, San Francisco, 1545 Divisadero Street, Suite 301, San Francisco, CA 94143 USA; 3 Vagelos College of Physicians and Surgeons, Columbia University Medical Center, 630 West 168th Street, New York, NY 10032 USA

**Keywords:** Testicular neoplasms, Leydig cell tumors, Sertoli cell tumors, Sex cord-gonadal stromal tumors, Testicular germ cell tumors

## Abstract

**Background:**

Testicular sex cord stromal tumors (SCSTs) are managed similarly to germ cell tumors (GCTs); however, few studies have directly compared outcomes between these tumor types. Using the National Cancer Database (NCDB), we sought to compare overall and stage-specific all-cause mortality (ACM) between SCSTs versus GCTs.

**Methods:**

NCDB was queried for patients diagnosed with SCSTs and GCTs between 2004 and 2013. Descriptive statistics were used to compare sociodemographic and clinical characteristics between groups. Univariable and multivariable Cox proportional hazards regression analyses were used to assess associations with ACM.

**Results:**

We identified 42,192 patients diagnosed with testicular cancer between 2004 and 2013, with 280 having SCSTs and 41,912 patients having GCTs. Median age for SCSTs and GCTs was 45 (interquartile range [IQR] 34–59) and 34 (IQR 27–43), respectively (*p* < 0.001). Median follow-up was 39 and 52 months, respectively. Overall, patients with SCSTs had greater risk of ACM compared to those with GCTs (HR 1.69, 95% CI 1.14–2.50). Private insurance, greater education, and fewer comorbidities were associated with reduced risk of ACM (*p* < 0.05 for all). Among those with stage I disease, tumor type was not associated with ACM on multivariable analysis. Among those with stage II/III disease, patients with SCSTs had increased risk of ACM compared to patients with GCTs (HR 3.29, 95% CI 1.89–5.72).

**Conclusions:**

Patients with advanced SCSTs had worse survival outcomes compared to those with advanced GCTs. These data suggest a need for further investigation to ascertain effective management recommendations for SCSTs.

## Background

Testicular cancer is a rare form of cancer, representing only 0.5% of new cancer cases in the United States (U.S.) [1]. It most commonly occurs in young adults, with 5.9 new cases per 100,000 men between 2012 and 2016 [1]. Testicular tumors can be further subclassified based on their cellular origin. Germ cell tumors (GCTs) account for roughly 95% of all testicular tumors and include seminomatous and nonseminomatous tumors (e.g., embryonal, mixed germ cell) [2]. Sex cord stromal tumors (SCSTs) make up the remaining 5% of testicular tumors and include Leydig cell tumors (LCTs) and Sertoli cell tumors (SCTs) [3].

SCSTs and GCTs differ greatly in their biological aggressiveness and survival outcomes. GCTs have an excellent prognosis, with a 99.7% 5-year disease-specific survival among those with Stage I tumors [4]. Patients with SCSTs appear to have worse survival outcomes, with the 5-year overall survival of patients with Stage I LCTs and SCTs being 91 and 77%, respectively [5]. This survival difference is multifactorial, with the inherent aggressiveness of SCSTs, differences in patient characteristics, and variable response to treatment likely contributing to this finding [6–8]. Prior studies focused on SCSTs alone, primarily small series and case studies, have demonstrated worse disease-specific and overall survival outcomes, but few studies have directly compared outcomes between SCSTs and GCTs [5, 9, 10]. These studies have been predominantly limited by the rarity of these tumors and few mortality events reported in large national cancer registries [5, 9].

Given that the American Urological Association (AUA) provides formal guidelines for the management of GCTs but none specific to SCSTs, the management of SCSTs primarily reflects that of GCTs [11]. Thus, a survival comparison between these tumor types is critical to identify whether a need for guidelines specific to SCSTs is warranted. Using a large, nationwide database of cancer patients, we sought to compare total and stage-specific all-cause mortality (ACM) outcomes between patients with GCTs and SCTSs with consideration of multiple sociodemographic and clinical characteristics.

## Methods

### Data source

The NCDB is a nationwide, facility-based cancer registry started in 1989 as a joint program between the Commission on Cancer of the American College of Surgeons and the American Cancer Society. It encompasses over 1500 institutions and contains over 70% of all cancer diagnoses in the U.S. and Puerto Rico [12, 13]. This study was approved by the Institutional Review Board at the University of California, San Francisco.

### Patient selection

Patients diagnosed between 2004 and 2013 were identified using the International Classification of Diseases for Oncology, Third Edition (ICD-O-3) codes for LCTs (8650), SCTs (8640), seminomas (9061), embryonal tumors (9070), and mixed germ cell tumors (9085). Diagnosis was determined from microscopic specimens, laboratory markers, imaging, or medical records from participating institutions. Those with unavailable staging information were excluded. Stage was defined by Clinical Stage Group or Clinical Pathological Group if the former was unavailable. Because of the low sample size of SCSTs, patients with stage II and stage III disease were collapsed into one group (stage II/III) for the survival analysis. We considered this a reasonable strategy, as both represent tumors that have spread beyond the testicle. Furthermore, this strategy has been performed in prior literature [5]. For the same reason, all forms of therapy received in addition to orchiectomy were grouped into an “adjuvant therapy” category. Of note, NCDB only reports first course treatments [14].

### Objectives and statistical analysis

The primary endpoint was ACM. Demographic, clinicopathologic, and overall survival outcome data was collected. Survival time was calculated as the time in months between diagnosis and either death or last follow-up contact. Patient characteristics were compared using Pearson’s chi-squared and Fischer’s exact tests for categorical variables and Wilcoxon rank-sum tests for non-parametric continuous variables. Survival curves were generated using the Kaplan-Meier plot method. Log-rank test and Cox proportional hazard regression was used to compare differences in survival among the entire cohort and on subgroup analysis by tumor stage (stage I and stage II/III). The other variables included in the multivariable models included tumor type, diagnosis year, race/ethnicity, insurance type, annual household income, percent in the patient’s ZIP code without a high school diploma, residential environment (metropolitan versus urban/rural), and Charlson-Deyo comorbidity score (CDS). As the stage and adjuvant therapy variables violated the proportionality assumption, analyses were stratified on these variables (STATA 15 command: *strata*) [15]. The results are presented as hazard ratios (HR) with 95% confidence intervals (CI). HRs for patients with unknown values are not reported but were included in analyses to avoid selection bias secondary to elimination of those patients. A *p*-value < 0.05 was considered statistically significant. Analyses were performed using STATA 15®.

## Results

### Sociodemographic and clinical characteristics between groups

Forty-two thousand one hundred ninety-two patients diagnosed with testicular cancer between 2004 and 2013 were identified (Table 1). There were 45 patients with SCTs and 235 patients with LCTs for a total of 280 patients with SCSTs. There were 26,394 patients with seminomas, 11,358 patients with embryonal tumors, and 4160 patients with mixed germ cell tumors for a total of 41,912 patients with GCTs. Median follow-up time was 39 months (interquartile range [IQR] 20–61) for SCSTs and 52 months (IQR 28–79) for GCTs. Compared to patients with GCTs, patients with SCSTs tended to be older (45 years IQR 34–59 versus 34 years IQR 27–43, *p* < 0.001). Higher proportions of patients with SCSTs were non-Hispanic Black (17% versus 3%) and were on government insurance (25% versus 13%) (all *p* < 0.001). Higher proportions of patients with SCSTs had one or more comorbidities (10% versus 6%, *p* = 0.001) and had stage I disease at diagnosis (93% versus 77%, *p* < 0.001). A considerably lower proportion of patients with SCSTs received adjuvant therapy in addition to orchiectomy (6% versus 56%, p < 0.001). Type of adjuvant therapy received in addition to orchiectomy by tumor type can be seen in Supplementary Table 1.

**Table 1:** Overall comparison of the sociodemographic and clinical characteristics of patients with SCSTs versus GCTs

Factor	Overall cohort	SCSTs	GCTs	***p***-value
N	42,192	280	41,912	
Age at diagnosis, median (IQR)	34 (27, 43)	45 (34, 59)	34 (27, 43)	< 0.001
Diagnosis year				0.002
2004–2005	8215 (20%)	48 (17%)	8167 (20%)	
2006–2007	8329 (20%)	39 (14%)	8290 (20%)	
2008–2009	8520 (20%)	50 (18%)	8470 (20%)	
2010–2011	8561 (20%)	81 (29%)	8480 (20%)	
2012–2013	8567 (20%)	62 (22%)	8505 (20%)	
Race/ethnicity				< 0.001
Non-Hispanic White	32,565 (77%)	174 (62%)	32,391 (77%)	
Non-Hispanic Black	1168 (3%)	48 (17%)	1120 (3%)	
Hispanic/Other	7727 (18%)	55 (20%)	7672 (18%)	
Unknown	732 (2%)	3 (1%)	729 (2%)	
Insurance				< 0.001
Uninsured	4786 (11%)	24 (9%)	4762 (11%)	
Private insurance	31,092 (74%)	178 (64%)	30,914 (74%)	
Medicaid/Medicare/other government insurance	5487 (13%)	70 (25%)	5417 (13%)	
Unknown	827 (2%)	8 (3%)	819 (2%)	
Income (per year)				0.42
Less than $38 k	5533 (13%)	45 (16%)	5488 (13%)	
$38 k-62,999	20,324 (48%)	125 (45%)	20,199 (48%)	
$63 k or greater	15,769 (37%)	106 (38%)	15,663 (37%)	
Unknown	566 (1%)	4 (1%)	562 (1%)	
Percent in ZIP code without a high school degree				0.81
21% or greater	6203 (15%)	42 (15%)	6161 (15%)	
7–20.9%	23,172 (55%)	159 (57%)	23,013 (55%)	
Less than 7%	12,287 (29%)	75 (27%)	12,212 (29%)	
Unknown	530 (1%)	4 (1%)	526 (1%)	
Residence				0.48
Metropolitan	35,048 (83%)	237 (85%)	34,811 (83%)	
Urban/rural	7144 (17%)	43 (15%)	7101 (17%)	
Charlson-Deyo comorbidity score				0.001
0	39,825 (94%)	252 (90%)	39,573 (94%)	
1 or more	2367 (6%)	28 (10%)	2339 (6%)	
Stage				< 0.001
Stage I	32,463 (77%)	259 (93%)	32,204 (77%)	
Stage II	5478 (13%)	9 (3%)	5469 (13%)	
Stage III	4251 (10%)	12 (4%)	4239 (10%)	
Treatment received				< 0.001
No orchiectomy	666 (2%)	2 (0.7%)	664 (2%)	
Orchiectomy alone	17,885 (42%)	260 (93%)	17,625 (42%)	
Orchiectomy + adjuvant therapy	23,585 (56%)	18 (6%)	23,567 (56%)	
Other/unknown	56 (0.1%)	0 (0%)	56 (0.1%)	
Last contact or death, months from diagnosis, median (IQR)	*N* = 37,716	*N* = 249	N = 37,467	
	52 (28, 79)	39 (20, 61)	52 (28, 79)	< 0.001
Time from diagnosis to death in months, median (IQR)	*N* = 1640	*N* = 27	*N* = 1613	
	20 (7, 43)	18 (7, 37)	20 (7, 43)	0.88

*GCTs* Germ cell tumors, *IQR* Interquartile range, *SCSTs* Sex cord stromal tumors

The aforementioned observations remained true when broken down by stage with a few exceptions (Table 2). There was no statistically significant difference in race/ethnic distribution or insurance status among those with advanced disease. Notably, among those with advanced disease, the magnitude of difference of the proportion of those with one or more comorbidities was greater among those with SCSTs (29% versus 7%, *p* < 0.001).

**Table 2:** Stage-specific comparison of the sociodemographic and clinical characteristics of patients with SCSTs versus GCTs

Factor	Stage I			Stage II/III		
	SCSTs	GCTs	p-value	SCSTs	GCTs	p-value
N	259	32,204		21	9708	
Age at diagnosis, median (IQR)	43 (34, 57)	34 (28, 43)	< 0.001	55 (42, 64)	33 (26, 42)	< 0.001
Diagnosis year			< 0.001			0.38
2004–2005	46 (18%)	6387 (20%)		2 (10%)	1780 (18%)	
2006–2007	33 (13%)	6416 (20%)		6 (29%)	1874 (19%)	
2008–2009	45 (17%)	6528 (20%)		5 (24%)	1942 (20%)	
2010–2011	75 (29%)	6487 (20%)		6 (29%)	1993 (21%)	
2012–2013	60 (23%)	6386 (20%)		2 (10%)	2119 (22%)	
Race/ethnicity			< 0.001			0.62
Non-Hispanic White	157 (61%)	25,125 (78%)		17 (81%)	7266 (75%)	
Non-Hispanic Black	47 (18%)	809 (3%)		1 (5%)	311 (3%)	
Hispanic/Other	52 (20%)	5672 (18%)		3 (14%)	2000 (21%)	
Unknown	3 (1%)	598 (2%)		0 (0%)	131 (1%)	
Insurance			< 0.001			0.087
Uninsured	169 (65%)	3411 (11%)		4 (19%)	1351 (14%)	
Private insurance	20 (8%)	24,575 (76%)		9 (43%)	6339 (65%)	
Medicaid/Medicare/other government insurance	62 (24%)	3605 (11%)		8 (38%)	1812 (19%)	
Unknown	8 (3%)	613 (2%)		0 (0%)	206 (2%)	
Income (per year)			0.38			0.68
Less than $38 k	40 (15%)	3981 (12%)		5 (24%)	1507 (16%)	
$38 k-62,999	115 (44%)	15,408 (48%)		10 (48%)	4791 (49%)	
$63 k or greater	100 (39%)	12,407 (39%)		6 (29%)	3256 (34%)	
Unknown	4 (2%)	408 (1%)		0 (0%)	154 (2%)	
Percent in ZIP code without a high school degree			0.69			0.75
21% or greater	37 (14%)	4450 (14%)		5 (24%)	1711 (18%)	
7–20.9%	147 (57%)	17,658 (55%)		12 (57%)	5355 (55%)	
Less than 7%	71 (27%)	9716 (30%)		4 (19%)	2496 (26%)	
Unknown	4 (2%)	380 (1%)		0 (0%)	146 (2%)	
Residence			0.42			0.51
Metropolitan	221 (85%)	26,877 (84%)		16 (76%)	7934 (82%)	
Urban/rural	38 (15%)	5327 (17%)		5 (24%)	1774 (18%)	
Charlson-Deyo comorbidity score			0.016			< 0.001
0	237 (92%)	30,544 (95%)		15 (71%)	9029 (93%)	
1 or more	22 (9%)	1660 (5%)		6 (29%)	679 (7%)	
Stage						0.21
Stage I	259 (100%)	32,204 (100%)		–	–	
Stage II	–	–		9 (43%)	5469 (56%)	
Stage III	–	–		12 (57%)	4239 (44%)	
Treatment			< 0.001			< 0.001
No orchiectomy	0 (0%)	30 (0.1%)		2 (10%)	634 (7%)	
Orchiectomy alone	250 (97%)	16,519 (51%)		10 (48%)	1106 (11%)	
Orchiectomy + adjuvant therapy	9 (4%)	15,626 (49%)		9 (43%)	7941 (82%)	
Other/unknown	0 (0%)	29 (0.1%)		0 (0%)	27 (0.3%)	
Last contact or death, months from diagnosis, median (IQR)	*N* = 229	*N* = 28,855	< 0.001	*N* = 20	*N* = 8612	0.002
	41 (22, 62)	53 (29, 80)		19 (8, 55)	47 (24, 75)	
Time from diagnosis to death, median (IQR)	*N* = 13	*N* = 747		*N* = 14	*N* = 866	
	23 (18, 43)	31 (14, 58)	0.78	11 (7, 21)	13 (4, 28)	0.96

*GCTs* Germ cell tumors, *IQR* Interquartile range, *SCSTs* Sex cord stromal tumors

### Survival analysis

On multivariable Cox proportional hazards regression of the overall group, patients with SCSTs had greater risk of ACM compared to those with GCTs (HR 1.68, 95% CI 1.13–2.49, *p* = 0.010) (Table 3) on multivariable analysis adjusting for stage and treatment via stratification. Compared to those with private insurance, those who were uninsured (HR 2.31, 95% CI 2.01–2.66, *p* < 0.001) or who had government insurance (HR 2.72, 95% CI 2.42–3.05, p < 0.001) had greater risk of ACM. Having one or more comorbidities also conferred a greater risk of ACM (HR 2.06, 95% CI 1.79–2.37, p < 0.001).

**Table 3:** Univariable and multivariable Cox proportional hazards regression analysis on the association between sociodemographic and clinical characteristics and mortality of the overall cohort

	Univariable HR (95% CI)	Multivariable^a^ HR (95% CI) – Overall
**Tumor type**		
GCTs	Ref.	Ref.
SCSTs	2.96 (2.03–4.33)***	1.68 (1.13–2.49)*
**Age (per 5-year increase)**	1.21 (1.19–1.23)***	1.18 (1.16–1.20)***
**Race/ethnicity**		
Non-Hispanic White	Ref.	Ref.
Non-Hispanic Black	1.80 (1.41–2.29)***	1.13 (0.89–1.45)
Hispanic/other	1.27 (1.13–1.43)***	1.14 (1.01–1.30)*
**Insurance**		
Private insurance	Ref.	Ref.
Uninsured	2.63 (2.29–3.01)***	2.32 (2.01–2.66)***
Medicaid/Medicare/other government insurance	4.33 (3.88–4.83)***	2.72 (2.42–3.05)***
**Income (per year)**		
< $38,000	Ref.	Ref.
$38,000–$62,999	0.71 (0.62–0.81)***	0.94 (0.82–1.09)
> $63,000	0.44 (0.38–0.50)***	0.76 (0.63–0.92)**
**Percent in ZIP code without a high school diploma**		
> 21%	Ref.	Ref.
7–20.9%	0.63 (0.56–0.71)***	0.83 (0.73–0.96)*
< 7%	0.43 (0.37–0.49)***	0.74 (0.61–0.90)**
**Residence**		
Metropolitan	Ref.	Ref.
Urban/rural	1.49 (1.33–1.67)***	1.13 (1.00–1.28)
**Charlson-Deyo comorbidity score**		
0	Ref.	Ref.
≥ 1	3.23 (2.82–3.70)***	2.06 (1.79–2.37)***

*CI* Confidence interval, *GCTs* Germ cell tumors, *HR* Hazard ratio, *IQR* Interquartile range, *SCSTs* Sex cord stromal tumors

*p < 0.05

**p < 0.01

***p < 0.001

^a^The following variables were included in the multivariable analysis: tumor type, age, diagnosis year, race/ethnicity, insurance, yearly income, percent in ZIP code without a high school diploma, residence, Charlson-Deyo comorbidity score

**Fig. 1:**
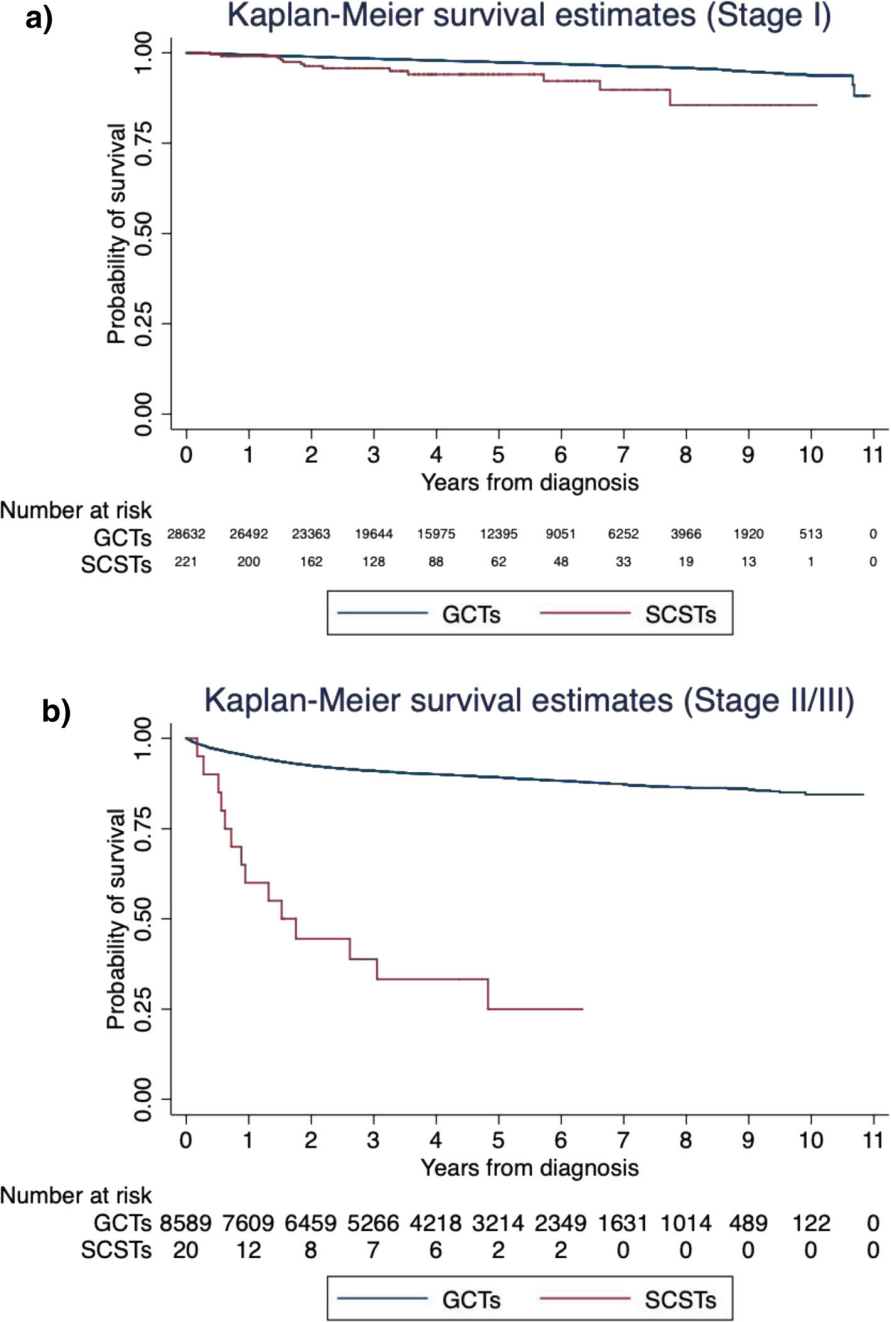
Kaplan-Meier survival estimates comparing all-cause mortality between patients with SCSTs versus GCTs among those with **a**) stage I disease and **b**) stage II/III disease

The Kaplan-Meier curves comparing overall survival outcomes between tumors types by stage can be seen in Fig. 1. At 1, 2, and 5 years, the overall survival rates for stage I SCSTs was 99% (95% CI 96–100%), 96% (95% CI 92–98%), and 94% (95% CI 89–97%), respectively and for stage I GCTs was 99% (95% CI 99–100%), 99% (95% CI 99–99%), and 97% (95% CI 97–98%), respectively (log-rank *p* < 0.001). Among those with stage I disease, tumor type was not associated with ACM on multivariable analysis (Table 4). High income (HR 0.74 among those making >$63,000/year compared to those making <$38,000/year, 95% CI 0.56–0.98, *p* = 0.032), was associated with lower ACM.

**Table 4:** Multivariable Cox proportional hazards regression analysis on the association between sociodemographic and clinical characteristics and mortality by stage

	Multivariable^a^ HR (95% CI) – Stage I	Multivariable^a^ HR (95% CI) – Stage II/III
**Tumor type**		
GCTs	Ref.	Ref.
SCSTs	1.06 (0.60–1.86)	3.28 (1.88–5.73)***
**Age (per 5-year increase)**	1.23 (1.20–1.26)***	1.13 (1.10–1.16)***
**Race/ethnicity**		
Non-Hispanic White	Ref.	Ref.
Non-Hispanic Black	1.18 (0.80–1.72)	1.13 (0.81–1.56)
Hispanic/other	1.14 (0.95–1.38)	1.12 (0.94–1.32)
**Insurance**		
Private insurance	Ref.	Ref.
Uninsured	2.58 (2.08–3.21)***	2.07 (1.72–2.50)***
Medicaid/Medicare/other government insurance	3.15 (2.64–3.75)***	2.31 (1.97–2.70)***
**Income (per year)**		
< $38,000	Ref.	Ref.
$38,000–$62,999	0.92 (0.74–1.15)	0.96 (0.79–1.16)
> $63,000	0.74 (0.56–0.98)*	0.77 (0.61–1.02)
**Percent in ZIP code without a high school diploma**		
> 21%	Ref.	Ref.
7–20.9%	0.87 (0.70–1.07)	0.80 (0.67–0.97)*
< 7%	0.80 (0.61–1.06)	0.68 (0.52–0.88)**
**Residence**		
Metropolitan	Ref.	Ref.
Urban/rural	1.18 (0.98–1.42)	1.09 (0.91–1.29)
**Charlson-Deyo comorbidity score**		
0	Ref.	Ref.
≥ 1	2.03 (1.64–2.51)***	2.03 (1.68–2.45)***

^a^The following variables were included in the multivariable analysis: tumor type, age, diagnosis year, race/ethnicity, insurance, yearly income, percent in ZIP code without a high school diploma, residence, Charlson-Deyo comorbidity score

At 1, 2, and 5 years, the overall survival rates for stage II/III SCSTs was 60% (95% CI 36–78%), 44% (95% CI 22–64%), and 25% (95% CI 8–47%), respectively and for stage II/III GCTs was 95% (95% CI 95–96%), 92% (95% CI 92–93%), and 89% (95% CI 88–90%) (log-rank *p* < 0.001). Among those with stage II/III disease, those with SCSTs had a statistically significantly increased risk of ACM (HR 3.28, 95% CI 1.88–5.73, *p* < 0.001) on multivariable analysis adjusting for treatment via stratification (Table 4). Percent of individuals in the patient’s ZIP code without a high school diploma (HR 0.67 for < 7% compared to > 21, 95% CI 0.52–0.89, *p* = 0.004) was associated with ACM.

## Discussion

Using a national registry of testicular cancer patients, we found that SCSTs conferred increased risk of ACM compared to patients with GCT. However, on multivariable subgroup analysis, this difference in overall survival was limited to those with advanced (stage II/III) disease. For these patients, the risk of ACM in the SCST group was more than three times greater than that of the GCT group. We also found that patients with SCSTs tended to be older and were more frequently of Black race.

The association between SCSTs and worse survival outcomes has been shown previously. Osbun et al. used the Surveillance, Epidemiology, and End Results (SEER) program database to estimate cancer-specific mortality (CSM) for various testicular tumor types. They found a lower overall CSM among patients with GCTs (2%) compared to those with LCTs (7%) or SCTs (32%, *p* < 0.001) [9]. They also reported an observation of higher unadjusted CSM rates in patients with SCSTs both in stage I and stage III disease. As with our population, higher proportions of SEER patients with SCSTs were older and Black; however, in contrast to our population, a higher proportion of SEER patients with SCSTs presented with advanced disease.

The finding that there is a greater risk of ACM with SCSTs versus GCTs among patients with advanced disease regardless of receipt of adjuvant therapy use indicates that management recommendations specific to SCSTs are needed. Currently, the AUA provides guidelines for the management of seminomatous and non-seminomatous GCTs but does not specifically address SCSTs [11]. Thus, much of the management reflects that of GCTs. An initial approach of radical resection for early stage disease has been shown to be effective [6, 16]. Featherstone et al. reported that, among 36 patients with SCSTs treated with orchiectomy alone, none progressed to metastatic disease after a minimum follow-up period of 2 years [16]. These results are consistent with the high overall survival among patients with stage I disease, and perhaps translation of management of SCSTs from that of GCTs is appropriate.

Using GCTs as a reference, our data suggests that patients with disseminated SCSTs do not experience the same relatively high overall survival rates as those with localized disease. Although surgical management via retroperitoneal lymphadenectomy may have modest efficacy in those with established nodal disease, traditional chemotherapy regimens used to treat GCTs like bleomycin, etoposide, and cisplatin (BEP) or vinblastine, ifosfamide, and cisplatin (VIP) have demonstrated a transient response in SCSTs [6–8]. Radiotherapy also appears to be ineffectual for these tumors [8, 17]. This lack of effectiveness signals an opportunity to investigate the potency of newer chemotherapeutics, hormonal therapies, and immunologic agents to improve outcomes for patients with this rare tumor type. For example, it has been demonstrated that glucocorticoid treatment may inhibit a hormone-mediated cell growth mechanism in LCTs that leads to tumor regression in murine models [18]. Imatinib, a selective tyrosine kinase inhibitor, has been shown to decrease viability of Leydig tumor cell lines [19]. Furthermore, with the advent of the use of genomics and precision medicine, future therapeutic regimens could potentially be tailored for these patients and may help improve treatment outcomes in patients with advanced disease [20]. Necchi et al. performed comprehensive genome profiling on the tumors of 10 patients with metastatic SCSTs and reported that, although uncommon, several tumors expressed targetable genomic alterations indicating the potential efficacy of cell-cycle, mTOR, hedgehog, and polymerase inhibitors [20]. A case-series by Calaway et al. reported that two patients with disseminated SCSTs had tumor genetic susceptibility testing following retroperitoneal lymph node dissection. At the time of publication, one patient was surviving on apalutamide for 4 months and another was on everolimus with slightly regressing metastatic disease for 12 months [21]. Finally, a step further would be to enhance methods for distinguishing malignant from benign LCTs for more directed adjuvant treatments.

This study is not without limitations. The inclusion of patients in this study assumes accurate designation of ICD-O-3 coding by providers and misclassification may bias the findings. This dataset also relies upon accurate reporting of histology, as there is no central pathology review. The NCDB has limited information on treatment specifics (e.g., type of chemotherapeutic agent), schedule and sequence of treatment cycles, and does not provide information on CSM. Despite these limitations, for those with advanced disease the differences between ACM and CSM rates are reduced. Additionally, the NCDB does not contain information that may influence quality of care received (e.g., hospital quality, surgeon experience, patient healthcare access/literacy, etc.); however, many factors that contribute to this (e.g., income, insurance, education) were controlled for in our analysis. As this study is a retrospective study based on an observational dataset, there are unmeasured confounders which could impact these findings. In addition, the small sample size of SCSTs limited multivariable comparisons of ACM between SCST subtypes, and it has been proposed that LCTs and SCTs may confer different degrees of ACM risk [5]. The small sample size also precluded analysis by adjuvant treatment type, which would help provide more actionable data on the most effective management strategy for these tumors. Along those same lines, our conclusion of survival differences between patients with advanced SCSTs versus advanced GCTs is based on just 21 SCST patients. Despite its comparison to a large group of GCT patients, this may limit the accuracy of our conclusions and warrants future studies with larger populations of SCST patients. The greater proportion of stage III patients in the SCST group versus the GCT group may have had an influence on survival; however, the absolute difference between groups was quite small. Additionally, the older age and greater proportion of patients with 1 or more comorbidity among those with advanced SCSTs could have been confounders in our survival analysis; however, these differences should have been controlled for in the multivariable model. Despite these limitations, this study presents novel data comparing testicular tumors types among a large number of patients with multiple sociodemographic and clinical variables considered, and its findings may have important implications in altering management practices.

## Conclusions

Among this cohort, when comparing SCSTs to GCTs, patients with stage II/III disease but not stage I disease was associated with a statistically significantly increased risk of ACM on multivariable analysis. Increased ACM in patients with advanced SCSTs indicates an imperative for targeted treatment regimens specific to this unique tumor type.

## Supplementary information

**Additional file 1: Table S1.** Comparison of therapy received in addition to orchiectomy among patients with SCSTs versus GCTs.

## Data Availability

The datasets generated and/or analysed during the current study are available in the National Cancer Database repository: https://www.facs.org/quality-programs/cancer/ncdb

## Abbreviations

ACM: All-Cause Mortality
AUA: American Urological Association
BEP: Bleomycin, Etoposide, and Cisplatin
CDS: Charleson-Deyo comorbidity score
CI: Confidence Interval
CSM: Cancer-Specific Mortality
GCT: Germ Cell Tumor
HR: Hazard Ratio
ICD-O-3: International Classification of Diseases for Oncology, Third Edition
IQR: Interquartile Range
LCT: Leydig Cell Tumor
SCT: Sertoli Cell Tumor
SCST: Sex Cord Stromal Tumor
SEER: Surveillance, Epidemiology, and End Results
U.S.: United States
VIP: Vinblastine, Ifosfamide, and Cisplatin
