# Correction to: A comparison of stage-specific all-cause mortality between testicular sex cordstromal tumors and germ cell tumors: results from the National Cancer Database

**DOI:** 10.1186/s12894-020-00672-9

**Published:** 2020-07-17

**Authors:** Kyle B. Zuniga, Samuel L. Washington, Sima P. Porten, Maxwell V. Meng

**Affiliations:** 1 Department of Urology, University of California, San Francisco, 550 16th Street, 6th Floor, San Francisco, CA 94158 USA; 2 Osher Center for Integrative Medicine, University of California, San Francisco, 1545 Divisadero Street, Suite 301, San Francisco, CA 94143 USA; 3 Vagelos College of Physicians and Surgeons, Columbia University Medical Center, 630 West 168th Street, New York, NY 10032 USA

**Correction to: BMC Urol (2020) 20:40**

**https://doi.org/10.1186/s12894-020-00609-2**

It was highlighted that the original article [1] contained the below errors in Tables 2, 3 and 4 and in the legends of Tables 3 and 4. This Correction article shows the correct Tables and legends.

1. In Table 2, for Stage I – SCSTs the number for “uninsured” and “privately insured” was swapped. The distribution should be uninsured = 20 (8%); privately insured = 169 (65%)
2. In Table 3, multivariable HR for Uninsured should read HR 2.31, 95% CI 2.01–2.66. Additionally, Urban/Rural should read HR 1.13, 95% CI 1.00–1.29. Among those with Stage II/III tumors, for percent of individuals in the patient’s ZIP code without a high school diploma, the less than 7% group should read HR 0.67, 95% CI 0.52–0.88. In Table 4, for stage II/III, HR for income >$63 k should read HR 0.79, 95% CI 0.61–1.02. Additionally, for stage II/III, for percent of individuals in the patient’s ZIP code without a high school diploma, the less than 7% group should read HR 0.67, 95% CI 0.52–0.88.
3. In Table 4, for stage II/III, HR for income >$63 k should read HR 0.79, 95% CI 0.61–1.02. Additionally, for stage II/III, for percent of individuals in the patient’s ZIP code without a high school diploma, the less than 7% group should read HR 0.67, 95% CI 0.52–0.88.
4. Table legend corrections: For Table 3, the definition of the abbreviation “IQR” is unnecessary. Table 4 was missing the following - CI = Confidence interval, GCTs = Germ cell tumors, HR = Hazard ratio, SCSTs = Sex cord stromal tumors, *p < 0.05, **p < 0.01, ***p < 0.001

**Table 2:** Stage-specific comparison of the sociodemographic and clinical characteristics of patients with SCSTs versus GCTs

Factor	Stage I			Stage II/III		
	SCSTs	GCTs	***p***-value	SCSTs	GCTs	p-value
N	259	32,204		21	9708	
Age at diagnosis, median (IQR)	43 (34, 57)	34 (28, 43)	< 0.001	55 (42, 64)	33 (26, 42)	< 0.001
Diagnosis year			< 0.001			0.38
2004–2005	46 (18%)	6387 (20%)		2 (10%)	1780 (18%)	
2006–2007	33 (13%)	6416 (20%)		6 (29%)	1874 (19%)	
2008–2009	45 (17%)	6528 (20%)		5 (24%)	1942 (20%)	
2010–2011	75 (29%)	6487 (20%)		6 (29%)	1993 (21%)	
2012–2013	60 (23%)	6386 (20%)		2 (10%)	2119 (22%)	
Race/ethnicity			< 0.001			0.62
Non-Hispanic White	157 (61%)	25,125 (78%)		17 (81%)	7266 (75%)	
Non-Hispanic Black	47 (18%)	809 (3%)		1 (5%)	311 (3%)	
Hispanic/Other	52 (20%)	5672 (18%)		3 (14%)	2000 (21%)	
Unknown	3 (1%)	598 (2%)		0 (0%)	131 (1%)	
Insurance			< 0.001			0.087
Uninsured	**20 (8%)**	3411 (11%)		4 (19%)	1351 (14%)	
Private insurance	**169 (65%)**	24,575 (76%)		9 (43%)	6339 (65%)	
Medicaid/Medicare/other government insurance	62 (24%)	3605 (11%)		8 (38%)	1812 (19%)	
Unknown	8 (3%)	613 (2%)		0 (0%)	206 (2%)	
Income (per year)			0.38			0.68
Less than $38 k	40 (15%)	3981 (12%)		5 (24%)	1507 (16%)	
$38 k-62,999	115 (44%)	15,408 (48%)		10 (48%)	4791 (49%)	
$63 k or greater	100 (39%)	12,407 (39%)		6 (29%)	3256 (34%)	
Unknown	4 (2%)	408 (1%)		0 (0%)	154 (2%)	
Percent in ZIP code without a high school degree			0.69			0.75
21% or greater	37 (14%)	4450 (14%)		5 (24%)	1711 (18%)	
7–20.9%	147 (57%)	17,658 (55%)		12 (57%)	5355 (55%)	
Less than 7%	71 (27%)	9716 (30%)		4 (19%)	2496 (26%)	
Unknown	4 (2%)	380 (1%)		0 (0%)	146 (2%)	
Residence			0.42			0.51
Metropolitan	221 (85%)	26,877 (84%)		16 (76%)	7934 (82%)	
Urban/rural	38 (15%)	5327 (17%)		5 (24%)	1774 (18%)	
Charlson-Deyo comorbidity score			0.016			< 0.001
0	237 (92%)	30,544 (95%)		15 (71%)	9029 (93%)	
1 or more	22 (9%)	1660 (5%)		6 (29%)	679 (7%)	
Stage						0.21
Stage I	259 (100%)	32,204 (100%)		–	–	
Stage II	–	–		9 (43%)	5469 (56%)	
Stage III	–	–		12 (57%)	4239 (44%)	
Treatment			< 0.001			< 0.001
No orchiectomy	0 (0%)	30 (0.1%)		2 (10%)	634 (7%)	
Orchiectomy alone	250 (97%)	16,519 (51%)		10 (48%)	1106 (11%)	
Orchiectomy + adjuvant therapy	9 (4%)	15,626 (49%)		9 (43%)	7941 (82%)	
Other/unknown	0 (0%)	29 (0.1%)		0 (0%)	27 (0.3%)	
Last contact or death, months from diagnosis, median (IQR)	*N* = 229	*N* = 28,855	< 0.001	*N* = 20	*N* = 8612	0.002
	41 (22, 62)	53 (29, 80)		19 (8, 55)	47 (24, 75)	
Time from diagnosis to death, median (IQR)	*N* = 13	*N* = 747		*N* = 14	*N* = 866	
	23 (18, 43)	31 (14, 58)	0.78	11 (7, 21)	13 (4, 28)	0.96

GCTs = Germ cell tumors, IQR = Interquartile range, SCSTs = Sex cord stromal tumors

**Table 3:** Univariable and multivariable Cox proportional hazards regression analysis on the association between sociodemographic and clinical characteristics and mortality of the overall cohort

	Univariable HR (95% CI)	Multivariable^1^ HR (95% CI) – Overall
**Tumor type**		
GCTs	Ref.	Ref.
SCSTs	2.96 (2.03–4.33)***	1.68 (1.13–2.49)*
**Age (per 5-year increase)**	1.21 (1.19–1.23)***	1.18 (1.16–1.20)***
**Race/ethnicity**		
Non-Hispanic White	Ref.	Ref.
Non-Hispanic Black	1.80 (1.41–2.29)***	1.13 (0.89–1.45)
Hispanic/other	1.27 (1.13–1.43)***	1.14 (1.01–1.30)*
**Insurance**		
Private insurance	Ref.	Ref.
Uninsured	2.63 (2.29–3.01)***	**2.31** (2.01–2.66)***
Medicaid/Medicare/other government insurance	4.33 (3.88–4.83)***	2.72 (2.42–3.05)***
**Income (per year)**		
< $38,000	Ref.	Ref.
$38,000–$62,999	0.71 (0.62–0.81)***	0.94 (0.82–1.09)
> $63,000	0.44 (0.38–0.50)***	0.76 (0.63–0.92)**
**Percent in ZIP code without a high school diploma**		
> 21%	Ref.	Ref.
7–20.9%	0.63 (0.56–0.71)***	0.83 (0.73–0.96)*
< 7%	0.43 (0.37–0.49)***	0.74 (0.61–0.90)**
**Residence**		
Metropolitan	Ref.	Ref.
Urban/rural	1.49 (1.33–1.67)***	1.13 (1.00–**1.29**)
**Charlson-Deyo comorbidity score**		
0	Ref.	Ref.
> 1	3.23 (2.82–3.70)***	2.06 (1.79–2.37)***

CI = Confidence interval, GCTs = Germ cell tumors, HR = Hazard ratio, SCSTs = Sex cord stromal tumors

*p < 0.05

**p < 0.01

***p < 0.001

^1^The following variables were included in the multivariable analysis: tumor type, age, diagnosis year, race/ethnicity, insurance, yearly income, percent in ZIP code without a high school diploma, residence, Charlson-Deyo comorbidity score

**Table 4:** Multivariable Cox proportional hazards regression analysis on the association between sociodemographic and clinical characteristics and mortality by stage

	Multivariable^1^ HR (95% CI) – Stage I	Multivariable^1^ HR (95% CI) – Stage II/III
**Tumor type**		
GCTs	Ref.	Ref.
SCSTs	1.06 (0.60–1.86)	3.28 (1.88–5.73)***
**Age (per 5-year increase)**	1.23 (1.20–1.26)***	1.13 (1.10–1.16)***
**Race/ethnicity**		
Non-Hispanic White	Ref.	Ref.
Non-Hispanic Black	1.18 (0.80–1.72)	1.13 (0.81–1.56)
Hispanic/other	1.14 (0.95–1.38)	1.12 (0.94–1.32)
**Insurance**		
Private insurance	Ref.	Ref.
Uninsured	2.58 (2.08–3.21)***	2.07 (1.72–2.50)***
Medicaid/Medicare/other government insurance	3.15 (2.64–3.75)***	2.31 (1.97–2.70)***
**Income (per year)**		
< $38,000	Ref.	Ref.
$38,000–$62,999	0.92 (0.74–1.15)	0.96 (0.79–1.16)
> $63,000	0.74 (0.56–0.98)*	**0.79** (0.61–1.02)
**Percent in ZIP code without a high school diploma**		
> 21%	Ref.	Ref.
7–20.9%	0.87 (0.70–1.07)	0.80 (0.67–0.97)*
< 7%	0.80 (0.61–1.06)	**0.67** (0.52–0.88)**
**Residence**		
Metropolitan	Ref.	Ref.
Urban/rural	1.18 (0.98–1.42)	1.09 (0.91–1.29)
**Charlson-Deyo comorbidity score**		
0	Ref.	Ref.
> 1	2.03 (1.64–2.51)***	2.03 (1.68–2.45)***

**CI = Confidence interval, GCTs = Germ cell tumors, HR = Hazard ratio, SCSTs = Sex cord stromal tumors**

*p **< 0.05**

**p **< 0.01**

***p **< 0.001**

^1^The following variables were included in the multivariable analysis: tumor type, age, diagnosis year, race/ethnicity, insurance, yearly income, percent in ZIP code without a high school diploma, residence, Charlson-Deyo comorbidity score

## References

[1] Zuniga KB (2020). A comparison of stage-specific all-cause mortality between testicular sex cordstromal tumors and germ cell tumors: results from the National Cancer Database. BMC Urol.

