# Supplementary material for: A comparison of stage-specific all-cause mortality between testicular sex cord stromal tumors and germ cell tumors: results from the National Cancer Database
Source: BMC Urol. 2020 Apr 17;20:40. doi: 10.1186/s12894-020-00609-2 (PMC7164279; doi:10.1186/s12894-020-00609-2)
Supplement: Supplementary file 1 — Additional file 1: Table S1. Comparison of therapy received in addition to orchiectomy among patients with SCSTs versus GCTs. [file 12894_2020_609_MOESM1_ESM.docx]

Supplementary Table 1: Comparison of therapy received in addition to orchiectomy among patients with SCSTs versus GCTs.

|  | SCSTs (N=278) | GCTs (N=41192) | p-value |
| --- | --- | --- | --- |
| Radiation |  |  | <0.001 |
| No | 270 (97%) | 29052 (71%) |  |
| Yes | 4 (1%) | 11479 (28%) |  |
| Unknown | 4 (1%) | 661 (2%) |  |
| Chemotherapy |  |  | <0.001 |
| No | 259 (93%) | 27475 (67%) |  |
| Yes | 11 (4%) | 12149 (30%) |  |
| Unknown | 8 (3%) | 1568 (4%) |  |
| Hormone therapy |  |  | 0.31 |
| No | 263 (95%) | 39497 (96%) |  |
| Yes | 3 (1%) | 259 (0.6%) |  |
| Unknown | 12 (4%) | 1436 (4%) |  |
| Immunotherapy |  |  | 0.83 |
| No | 275 (99%) | 40522 (98%) |  |
| Yes | 0 (0%) | 15 (<0.01%) |  |
| Unknown | 3 (1%) | 655 (2%) |  |
| RPLND |  |  | 0.80 |
| No | 247 (89%) | 36716 (89%) |  |
| Yes | 30 (11%) | 4162 (10%) |  |
| Unknown | 1 (0.4%) | 314 (0.8%) |  |

GCTs = Germ cell tumors, RPLND = Retroperitoneal lymph node dissection, SCSTs = Sex cord stromal tumors
